# Evaluation of a screening and isolation strategy to curb carbapenem-resistant gram-negative bacteria in Hanoi, Vietnam: a pragmatic before-after study

**DOI:** 10.1186/s12879-025-11736-2

**Published:** 2025-10-09

**Authors:** Nguyen Quang TOAN, Buy Tien SY, Hoang Bao LONG, Corinne Tamames, Vu Viet SANG, Nguyen Van TRONG, Pham Dang HAI, Ngo Dinh TRUNG, Nguyen Van TUYEN, Dang Viet DUC, Mai Hong BANG, Le Huu SONG, Nguyen Thi Kim PHUONG, Jérôme Robert

**Affiliations:** 1 Department of Infection Control, 108 Military Central Hospital, Hanoi, Vietnam; 2 Department of Microbiology, 108 Military Central Hospital, Hanoi, Vietnam; 3 College of Health Sciences, Vin University, Hanoi, Vietnam; 4 Infection Control Unit, Pitié-Salpêtrière Hospital, APHP. Sorbonne Université, Paris, France; 5 Department of Respiratory Diseases, 108 Military Central Hospital, Hanoi, Vietnam; 6 Internal Medicine ICU, 108 Military Central Hospital, Hanoi, Vietnam; 7 Surgical and Transplantation ICU, 108 Military Central Hospital, Hanoi, Vietnam; 8 Stroke Department, 108 Military Central Hospital, Hanoi, Vietnam; 9 Cardiological ICU, 108 Military Central Hospital, Hanoi, Vietnam; 10 108 Military Central Hospital, Hanoi, Vietnam; 11 INSERM, CNRS, Centre d’immunologie et de maladies infectieuses - CIMI, Sorbonne-Université, Paris, France

**Keywords:** Carbapenemase, Hand hygiene, ICU, Isolation precaution, Multidrug resistance, Screening, Vietnam

## Abstract

**Background:**

Carbapenem resistance among gram-negative bacilli is a major threat worldwide, particularly in Vietnam. The study aimed to evaluate the magnitude of carbapenem resistance in gram-negative bacilli in intensive care units (ICU) in Vietnam and evaluate the impact of a screening and isolation strategy on their epidemiology.

**Methods:**

A before-after study was performed where patients were screened for digestive carriage of carbapenem-resistant and carbapenemase-producing gram-negative (CRGN) bacilli at ICU admission and weekly thereafter during a reference period and an intervention period. The intervention consisted of the implementation of isolation precautions for carriers throughout their ICU hospitalization.

**Results:**

The proportion of CRGN digestive carriers at admission was 31.1% in the reference period and 32.6% in the intervention period. The characteristics associated with admission carriage were antibiotic treatment before ICU admission (OR 3.5) and a prior history of hospitalization (OR 1.7). The acquisition rate at ICU discharge was lower in the intervention period (2.79/100 patient-days) than in the reference period (6.85/100 patient-days; *P* < 0.001). The intervention period was the only characteristic associated with a lower hazard of CRGN bacilli acquisition during the ICU stay (hazard ratio: 0.27, 95%CI 0.21–0.35) in a multivariable Cox model. In addition, the overall compliance with hand hygiene increased significantly in the intervention period (80.0%) as compared to the reference period (52.8%, *P* < 0.001).

**Conclusions:**

The proportions of carriers of CRGN bacilli at ICU admission and during the ICU stay were very high. The intervention, which included active screening and isolation precautions, along with increased hand hygiene, proved to be efficient in decreasing the CRGN bacilli acquisition rate.

**Supplementary Information:**

The online version contains supplementary material available at 10.1186/s12879-025-11736-2.

## Introduction

Antimicrobial resistance (AMR) is a major threat to public health worldwide. In 2019, it was estimated that 5 million deaths were associated with AMR, including 1.3 million deaths directly attributable to AMR [1, 2]. A recent study from the Global Burden of Diseases Collaborator network forecasted that, in 2050, more than 8 million deaths associated with AMR could occur, with 1.9 million attributable to AMR [3]. Based on current data and forecasting, the death rate attributable to AMR in Southeast Asia (SEA) is expected to increase by 67% from 2021 to 2050 ^3^. The three leading carbapenem-resistant gram-negative (CRGN) species in the super-region including SEA, East Asia, and Oceania are *Acinetobacter baumannii*, *Pseudomonas aeruginosa*, and *Klebsiella pneumoniae* [3].

Numerous surveillance studies on AMR have been conducted in SEA, particularly in Vietnam. The Vinares project [4] was initiated in 2012 and gathered data from the hospital setting for the 2016–2017 period. At that time, 27%, 45%, and 79% of *K. pneumoniae*, *P. aeruginosa*, and *A. baumannii* isolates, respectively, were reported to be resistant to carbapenems. Other recent studies have confirmed the high burden of CRGN species and the spread of carbapenemase genes in Vietnamese hospitals [5–8].

The Vietnamese Ministry of Health issued an action plan for the prevention and control of antimicrobial resistance in healthcare for the period 2024–2025. The overall goal of the plan is to slow the progression of antimicrobial resistance and prevent and control the spread of antimicrobial-resistant microorganisms and infectious diseases while ensuring the continuous availability and appropriate use of antimicrobials to treat infectious diseases effectively in humans and animals.

Hospitals in Vietnam, as in most other countries, focused their infection control activities on preventing respiratory infections and improving hand hygiene during and immediately following the COVID-19 pandemic. More recently, because antibiotic resistance is of major concern in Vietnam, especially in healthcare settings, Military Central Hospital 108 (MCH-108) planned to reinforce its strategy against hospital-acquired infections related to multidrug-resistant organisms (MDROs). Overall, a retrospective analysis of available local data from the hospital laboratory revealed that carbapenem resistance among all Enterobacterales increased from 17.2% to 30.6% from 2020 to 2023 (personal data). Similarly, the proportion of carbapenem-resistant *P. aeruginosa* increased from 37.2% to 45.0% within the same time frame (personal data). Nevertheless, the interpretation of these data should be taken with caution because it is likely to be hampered by a low level of request for clinical samples [9], and no tests are routinely performed to detect carbapenemase genes. Hence, as a first step, the hospital sought to evaluate the magnitude of antibiotic resistance, especially that linked to carbapenemase production, and its characteristics in MCH-108 intensive care units (ICU). A screening and isolation policy was subsequently implemented to curb cross-transmission of CRGN bacilli in the ICU. We present the current characteristics of CRGN carriage at ICU admission and evaluate the impact of the new policy on the epidemiology of CRGN bacilli in the participating ICU.

## Materials and methods

### Population

MCH-108 is a 2000-bed healthcare facility in central Hanoi, Vietnam. It serves the local population as well as military forces and is also a national tertiary care referral center for many specialties, therefore admitting patients from facilities throughout the country. The hospital has five distinct ICU. A surgical intensive care unit and transplantation (ICU-1) with 8 physicians and 30 nurses working in 12-hour shifts that admits circa 1200 patients per year. A medical and clinical toxicology ICU (ICU-2) with 8 physicians and 25 nurses working in 12-hour shifts that admits circa 1100 patients per year. A neurocritical care ICU (ICU-3) with 7 physicians and 24 nurses that admits circa 650 patients per year. A cardiovascular ICU (ICU-4) with 6 physicians and 22 nurses that admits circa 650 patients per year. A respiratory and infectious diseases ICU (ICU-5) with 7 physicians and 24 nurses that admits circa 550 patients per year. All 3 latter ICU have one shift from 7:30 to 17:00 and one from 17:00 to 7:30. All ICU have 15 beds, except the infectious diseases ICU, which has 10 beds. All ICU follow the same layout: one multiple-bed room accommodating 2 to 4 patients, with the remaining rooms being single-bed rooms. Each ICU admits patients according to its respective specialty. All patients admitted to one of the five distinct ICU of the MCH-108 hospital during the two study periods were included in the study pending their consent to participate.

### Design

We performed a before-after study including all patients admitted to one of the five distinct ICU of the MCH-108 hospital.

During the first 7-month period (“before” or reference period – September 2022 to March 2023), all patients admitted to the participating ICU were screened for digestive carriage of CRGN bacteria by using rectal swabs collected on admission and then once a week and at discharge. No other changes in patient management were implemented.

This reference period was followed by a 5-month training period (April 2023–August 2023), during which the staff of the five ICU was trained for isolation precautions and cohorting. In addition, reminders on hand hygiene with alcohol hand rubs were given. No data were recorded during this training period.

During the following 7-month period (“after” or intervention period, September 2023–March 2024), patients admitted to the ICU were systematically placed under preemptive isolation precautions on admission until the results of the admission CRGN screening swab culture were obtained. Then, isolation precautions were implemented for all patients harboring carbapenemase-positive Enterobacterales (CPE), carbapenemase-positive *P. aeruginosa* (CPPA), and carbapenem-resistant *A. baumannii* (CRAB) in a screening swab or a clinical sample. The policy was to place carriers of CRGN bacilli in a single room or cohort in double-bed rooms when necessary. Patients negative on admission were managed as during the reference period with standard precautions. Antibiotic use, the process of the diagnosis, and the management of clinical infections were not changed during the intervention period. The environment of the patient was cleaned twice a day for carriers, whereas it was cleaned once a day for noncarriers, following standard care as in the reference period.

When an isolate included in the program was identified during the intervention period, both the clinician in charge and the infection control team were informed without delay by the microbiology laboratory.

In addition, the following data were retrospectively collected from patients’ electronic files for the reference and intervention periods: age, sex, source of admission, antibiotic treatment, hospital-acquired infections, invasive devices, and microbiological data. For analysis, antibiotics were grouped as follows: cephalosporins (cefoxitin and third-generation cephalosporins), penicillins (ampicillin, amoxicillin, ticarcillin, and piperacillin), beta-lactams combined with inhibitors (piperacillin and tazobactam, ticarcillin and clavulanate), carbapenems, colistin, aminoglycosides, fluoroquinolones, newer antibiotics (ceftolozane and tazobactam, ceftazidime and avibactam, cefoperazone and sulbactam), and other remaining antibiotics.

Finally, during both study periods, the infection control staff monitored monthly alcohol-based hand rub (ABHR) consumption and hand hygiene (HH) compliance via direct observation. The compliance with isolation precautions during the intervention period was also monitored via regular direct observations.

### Bacteriological analysis

The hospital has a local microbiology laboratory with technicians and biologists dedicated to this activity. Rectal swabs were plated on ChromID^®^ Carba plates (bioMérieux, Marcy-l’Etoile, France). After incubation, growing colonies were identified by using either VITEK^®^ MS mass spectrometry or VITEK^®^−2 GN cards (both from bioMérieux, France). Antibiotic susceptibility tests were performed by using VITEK^®^ AST-N204 cards (bioMérieux, France). Carbapenemase production was ascertained for carbapenem-resistant Enterobacterales or *P. aeruginosa* isolates by using the NG-test CARBA 5^®^ (NG Biotech, Guipry, France) following the manufacturer’s operating manual. For *A. baumannii* isolates, no confirmatory tests were used because none were available in the country at that time. Hence, all the CRABs were included in the study.

### Definitions

Patients were considered carriers on the date of the first screening swab or clinical sample displaying a CRGN of interest. Carriage on admission was defined when the date of the first positive screening swab or a positive clinical sample fell ≤ 48 h after ICU admission. CRGN bacilli acquisition during ICU stay was defined by a CRGN-positive sample following a negative admission test and no positive clinical sample on admission.

Exposure to antibiotics and invasive devices was recorded before the first CRGN-positive sample or until ICU discharge for those remaining free of CRGN bacilli.

### Statistical analysis

Descriptive analysis was conducted via conventional methods. Categorical variables are presented as counts (percentages), and quantitative variables are presented as the means (standard deviations) or medians (interquartile ranges, IQRs). Differences between groups were tested by using the chi-square test or Fisher’s exact test for categorical variables and *t-tests*, analysis of variance (ANOVA), or the Kruskal-Wallis test for quantitative variables, where appropriate.

Multivariable logistic regression was employed to determine the factors independently associated with being a CRGN carrier at admission. The covariates included in the logistic model were those with *P* < 0.20 in the univariable analysis and variables deemed clinically relevant regardless of the results of the univariable analysis.

To compare CRGN acquisition between the two periods, in addition to the cumulative incidence, we calculated the incidence rate of CRGN acquisition, where the numerator was the number of new CRGN acquisitions, and the denominator was the total person-days until the development of CRGN acquisition or the end of follow-up. We compared the overall incidence rates between the two periods (intervention versus reference) and stratified by department, sex, age group (≤ 65 or > 65), origin before ICU, and history of previous hospitalization.

We also estimated the probability of developing CRGN via the Kaplan‒Meier method. Multivariable Cox proportional hazards models were performed to estimate the difference in hazard between the two periods, adjusting for department, sex, age group, history of previous hospitalization, antibiotic use before the ICU, and antibiotic use during the ICU. For sensitivity analyses, we added an interaction term between the period and department to examine the effect of heterogeneity among departments; the significance of the interaction term was checked by using the likelihood ratio test for a nested model (the noninteraction model was nested within the interaction model).

The data were cleaned and analyzed via Stata 18/BE (StataCorp, College Station, TX) and the R Software version 4.4.0 (R Foundation for Statistical Computing). Two-sided *P* < 0.05 was considered statistically significant.

### Ethical issues

The ethical committee for biomedical research of MCH-108 granted ethical clearance for this study (CRE 07/06/2020). This committee is approved by the Ministry of Defense of Vietnam and follows the Declaration of Helsinki. All data were anonymously extracted from patients’ files. Informed consent to participate was obtained from all of the patients included in the study.

## Results

### Patient characteristics and carriage at ICU admission

A total of 450 and 448 patients were included in the reference and intervention periods, respectively. The characteristics of the patients in both periods are displayed in Table 1. The distributions of the patients in the five ICU were slightly different between the two periods, with more patients admitted to ICU-2 and ICU-5 during the intervention period than during the reference period. Patients from other healthcare institutions were more common in the intervention period (46.2% vs. 35.3%), whereas patients from home (9.1%) or other wards from MCH-108 (36.9%) were more common in the reference period than in the intervention period (4.7% and 29.9%, respectively). Finally, compared with those in the reference period, more patients were treated with antibiotics (96.2% vs. 91.3%, *P* = 0.003) and had indwelling urinary catheters (96.9% vs. 89.3%, *P* < 0.001). The total number of days in ICU was higher in the intervention period than in the reference period (median 13 vs. 12 days, *P* = 0.005). Interestingly, there was no difference in the proportion of CRGN carriers admitted to the ICU during either period (31.1% vs. 32.6%, *P* = 0.635). During the reference period and the intervention period, most carriers at admission harbored *K. pneumoniae* (25.8% and 26.1%, respectively). The remaining positive patients harbored *E. coli* (6.7% and 8.7%, respectively) and *A. baumannii* (6.4% and 5.1%, respectively). Among the carbapenemase-positive Enterobacterales isolated at ICU admission in the reference and intervention periods, 46.4% and 41.7% were positive for OXA-48-like genes, 44.4% and 32.7% for KPC genes, and 44.4% and 58.9% for NDM genes, respectively. Approximately one-third of the Enterobacterales isolates were positive for at least two different carbapenemase genes. All *P. aeruginosa* strains isolated on admission on ChromID^®^ carba plates were NDM positive. The proportion of carriers at ICU discharge was greater in the reference period (74.7%) than in the intervention period (56.7%, *P* < 0.001).

**Table 1:** Characteristics (n, %) of the patients in the reference and intervention periods

Characteristic	Reference	Intervention	*P* value
	*n* = 450 (100)	*n* = 448 (100)	
Qualitative variables	*n* (%)	*n* (%)	
Gender (male)	316 (70.2)	292 (65.2)	0.106
ICU number			0.002
1	107 (23.8)	88 (19.6)	
2	103 (22.9)	119 (26.6)	
3	102 (22.7)	102 (22.8)	
4	78 (17.3)	48 (10.7)	
5	60 (13.3)	91 (20.3)	
Patients’ origin before ICU			0.001
Home	41 (9.1)	21 (4.7)	
Emergency room	84 (18.7)	86 (19.2)	
Other healthcare institution	159 (35.3)	207 (46.2)	
Other wards of MCH-108	166 (36.9)	134 (29.9)	
Prior hospitalization	341 (75.8)	351 (78.4)	0.360
Antibiotic before ICU	203 (45.1)	259 (57.8)	< 0.001
During ICU stay			
Antibiotic treatment	411 (91.3)	431 (96.2)	0.003
Mechanical ventilation	373 (82.9)	374 (83.5)	0.812
Intravenous catheter	293 (65.1)	310 (69.2)	0.192
Urinary catheter	402 (89.3)	434 (96.9)	< 0.001
CRGN carriers* on admission, including	140 (31.1)	146 (32.6)	0.635
* K. pneumoniae*	116 (25.8)	117 (26.1)	0.908
* E. coli*	30 (6.7)	39 (8.7)	0.251
other Enterobacterales	7 (1.6)	12 (2.7)	0.242
* P. aeruginosa*	14 (3.1)	22 (4.9)	0.169
* A. baumannii*	29 (6.4)	23 (5.1)	0.401
CRGN positive on ICU discharge	336 (74.7)	254 (56.7)	< 0.001
Quantitative variable	Median (25–75%)	Median (25–75%)	
Age (years)	66.0 (53–76)	66 (50–74)	0.146
Days in hospital before ICU	2 (1–5)	1 (1–5)	0.849
Total days in ICU	13 (10–17)	13 (10–18)	0.006

* Carbapenem-resistant gram-negative bacilli; patients may carry more than one type of species

Combining both periods (Table 2), patients admitted to ICU with CRGN organisms were more likely than those free of CRGN bacilli on ICU admission to come from other wards of MCH-108 (45.8% vs. 27.6%), to have a prior history of hospitalization (90.6% vs. 70.8%, *P* < 0.001) and a longer hospital stay before ICU admission (median 5 days vs. 1 day, *P* < 0.001), to have received antibiotics before ICU admission (74.8% vs. 40.5%, *P* < 0.001) and during ICU stay (99.3% vs. 91.2%, *P* < 0.001). They were also more likely to be admitted to ICU-2 (31.8% vs. 21.4%) and less likely to be admitted to ICU-3 (16.4% vs. 25.7%). In a multivariable logistic regression analysis (Table 3), factors independently associated with the risk of being a carrier at ICU admission during both periods were an antibiotic treatment regimen before ICU admission (odds ratio 3.5, 95% confidence interval – 95%CI 2.4-5.0) and a prior history of hospitalization (odds ratio - OR 1.7, 95%CI 1.04–2.9). Admission to ICU-3 was inversely linked to CRGN carriage on admission (OR 0.6, 95%CI 0.4–0.8). When each period was analyzed separately, antibiotic treatment before ICU admission remained associated with a higher risk of carriage on admission, but prior history of hospitalization did not remain associated to carriage (Table 3).

**Table 2:** Characteristics (*n*, %) of patients with and without CRGN bacilli* at ICU admission during both periods

Characteristic	CRGN-positive	CRGN-negative	*P* value
Qualitative variables	*n* = 286 (100.0)	*n* = 612 (100.0)	
Gender (male)	191 (66.8)	417 (68.1)	0.686
ICU number			0.001
1	70 (24.5)	125 (20.4)	
2	91 (31.8)	131 (21.4)	
3	47 (16.4)	157 (25.7)	
4	27 (9.5)	99 (16.2)	
5	51 (17.8)	100 (16.3)	
Patients’ origin before ICU			< 0.001
Home	8 (2.8)	54 (8.8)	
Emergency room	30 (10.5)	140 (22.9)	
Other healthcare institution	117 (40.9)	249 (40.7)	
Other wards of MCH-108	131 (45.8)	169 (27.6)	
Prior hospitalization	259 (90.6)	433 (70.8)	< 0.001
Antibiotic before ICU admission	214 (74.8)	248 (40.5)	< 0.001
During ICU stay			
Antibiotic treatment	284 (99.3)	558 (91.2)	< 0.001
Mechanical ventilation	256 (89.5)	491 (80.2)	0.001
Intravenous catheter	222 (77.6)	381 (62.3)	< 0.001
Urinary catheter	280 (97.9)	556 (90.9)	< 0.001
Quantitative variable	Median (25–75%)	Median (25–75%)	
Age (years)	67 (53–75)	65 (51–75)	0.599
Days in hospital before ICU	5 (1–10)	1 (0–3)	< 0.001
Total days in ICU	13 (10–18)	12 (9–17)	0.003

* Carbapenem-resistant gram-negative bacilli

**Table 3:** Factors independently associated with the risk of being a carrier of CRGN bacilli* at ICU admission after multivariable analysis

Characteristic	Both periods		Reference period		Intervention period	
	OR [95%CI]	*P* value	OR [95%CI]	*P* value	OR [95%CI]	*P* value
Antibiotic before ICU	3.5 [2.4-5.0]	< 0.001	7.0 [4.5–10.9]	< 0.001	2.8 [1.8–4.5]	< 0.001
Prior hospitalization	1.7 [1.04–2.9]	0.035				
Admission in ICU 2			2.4 [1.5–3.9]	< 0.001		
Admission in ICU 3	0.6 [0.4–0.8]	< 0.001			0.6 [0.4–0.9]	0.026

* Carbapenem-resistant gram-negative bacilli

### Change in CRGN acquisition after policy implementation

The characteristics of patients free of CRGN bacilli at ICU admission were slightly different between the reference and intervention periods (see Supplementary Table S1). Indeed, compared with those in the reference period, those free of CRGN bacilli at ICU admission during the intervention period were more likely to be hospitalized in ICU-2 (24.8% vs. 18.1%) and ICU-5 (19.2% vs. 13.6%); to come from other healthcare institutions (46.4% vs. 35.2%); to have invasive devices within the ICU (95.7% vs. 86.8%, *P* < 0.001), especially urinary catheters (95.7% vs. 86.1%, *P* < 0.001); to be treated with antibiotics before ICU admission (50.0 vs. 31.3%, *P* < 0.001); to have received antibiotics within the ICU (94.4% vs. 88.1%, *P* = 0.006); and for a longer duration (median 9 days vs. 7 days, *P* < 0.001). Finally, the proportion of hospital-acquired infections among negative carriers on admission was lower in the intervention period (20.5%) than in the reference period (39.7%, *P* < 0.001).

**Fig. 1:**
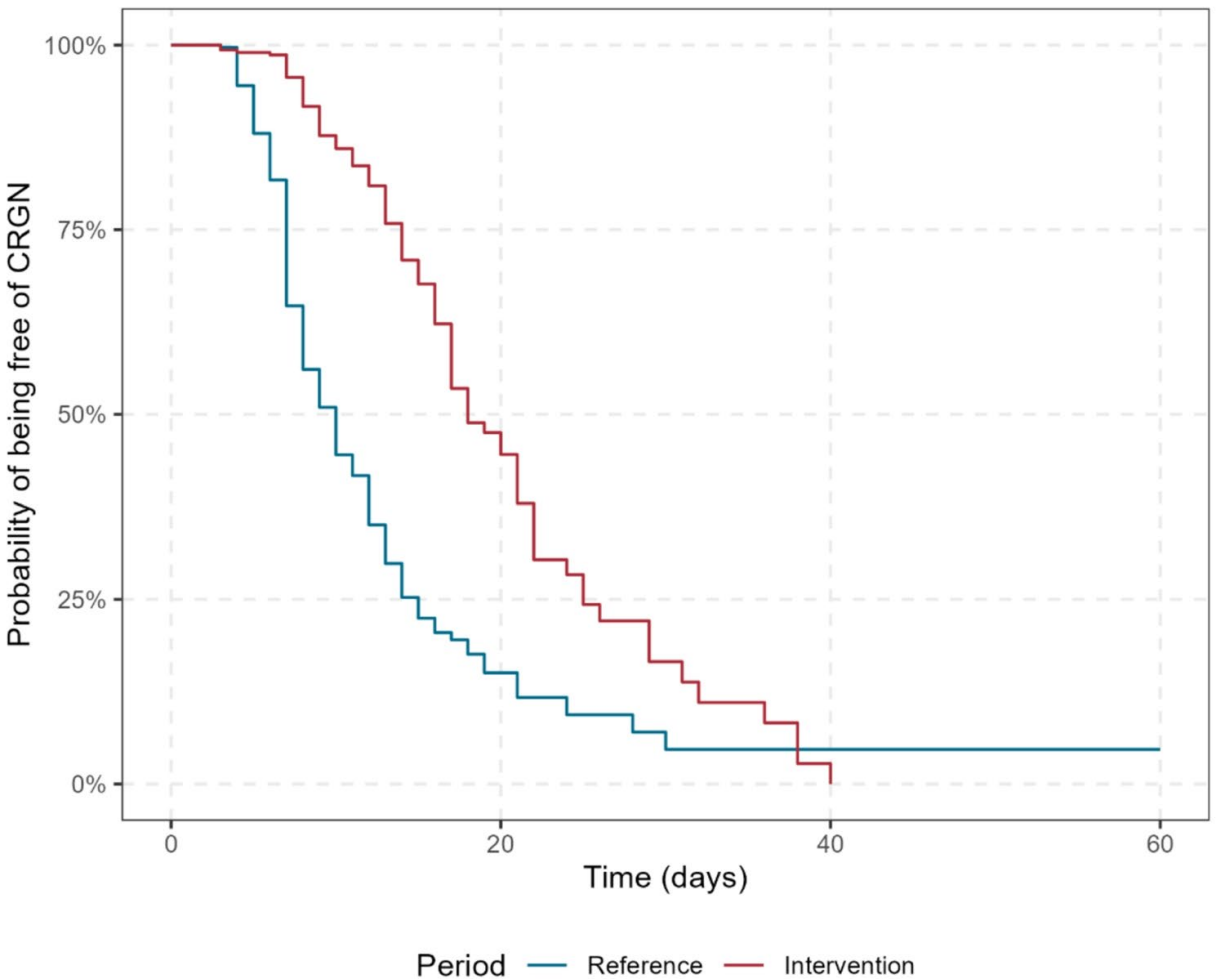
Survival curves presenting the probability of being free of carbapenem-resistant gram-negative (CRGN) bacilli, as estimated by the Kaplan‒Meier method, between the reference and intervention periods

Among the patients free of CRGN bacilli at ICU admission, the proportion of CRGN carriers at ICU discharge and the acquisition rate were significantly lower in the intervention period (35.5% and 2.79/100 patient-days) than in the reference period (63.2% and 6.85/100 patient-days, respectively; *P* < 0.001). The differences in incidence density rates were consistent in the subgroup analyses of the participating ICU, sex, age groups, origins before admission, and history of previous hospitalization (see Supplementary Table S2). *K. pneumoniae* (alone or combined with another species) represented 60.2% and 68.3% (*P* = 0.169) of the CRGN bacilli acquired within ICU during the reference and intervention periods, respectively. The median time to acquisition was longer in the intervention period (12 days, 95%CI 11–12) than in the reference period (8 days, 95%CI 8–8) (Fig. 1). According to a multivariable Cox model including periods, ICU, prior history of hospitalization, antibiotics before and within the ICU, and the total duration of the antibiotic regimen within the ICU, only the intervention period was associated with a lower hazard of CRGN bacilli acquisition during the ICU stay (hazard ratio- HR: 0.27, 95%CI 0.21–0.35). The total days with antibiotics within the ICU also had an inverse association with CRGN bacilli acquisition (HR: 0.97, 95%CI 0.96–0.98). These findings were consistent among the different multivariable Cox models in the sensitivity analysis. (data not shown).

Notably, indicators recorded for monitoring hygiene compliance were significantly different between the two periods. The mean number of ABHRs per patient and per day was greater in the intervention period (mean 43.8 rubs) than in the reference period (mean 34.3 rubs; *P* < 0.001). Compared with the reference period, HH compliance, as observed by the infection control team, was greater in the intervention period for overall compliance (80.0% vs. 52.8%, *P* < 0.001), before (78.1% vs. 45.3%, *P* < 0.001), and after (76.0% vs. 52.3%, *P* < 0.001) patient care. In addition, during the intervention period, an information sign was posted on the rooms of the CRGN bacilli carriers in 92.1% of the cases, and carriers were placed in single rooms or cohorted in 92.4% of the cases.

## Discussion

We report herein the results of an infection control intervention to curb CRGN bacilli in Vietnamese ICU. The intervention consists of a combination of patient screening on admission by using selective chromogenic media and, subsequently, during the ICU stay, of isolation precautions for CRGN bacilli carriers. We showed that, during the intervention period, the acquisition rate of CRGN organisms decreased by 46% and that being hospitalized during the intervention period was independently associated with a lower risk of acquisition of CRGN bacilli.

The study was conducted during a period of high prevalence of CRGN carriage. Indeed, during both study periods, almost one-third of the patients were CRGN carriers at ICU admission. This high proportion of CRGN carriage is consistent with previous reports from Vietnam [8, 10]. In addition, the circulation of carbapenemase genes among Enterobacterales, especially *K. pneumoniae*, and CRAB have been reported among ICU patients in Vietnam, including those admitted to the ICU [6, 11, 12]. However, these reports rarely evaluate the prevalence rate of colonization or infection by carbapenemase-producing organisms on ICU admission because the time of sampling is frequently missing. Moreover, information on the sampling strategy for digestive carriage or for the diagnosis of infection, as well as the management of duplicate isolates, is often overlooked, preventing proper evaluation of biases [13]. Finally, in low- and middle-income countries, requests for microbiological tests by clinicians tend to be reserved for the most difficult-to-treat cases [9, 14], resulting in biased levels of antibiotic resistance toward higher rates. Our report provides new and recent unbiased data regarding the high burden of CRGN bacilli in Vietnam because we used a systematic screening strategy on patient admission and discharge, although we did not evaluate infection rates. As expected, we showed that a prior history of hospitalization and a previous antibiotic treatment regimen were the major factors associated with the carriage of CRGN organisms at ICU admission. These findings have been previously reported in Vietnam [8] or elsewhere [15] and should drive hygiene intervention to prevent further cross-transmission within ICU.

Our study supports the efficacy of CRGN bacterial screening on CHROMID^®^ CARBA media combined with contact precautions to curb CRGN bacilli in a highly endemic setting. Indeed, being hospitalized during the intervention period was associated with a 61.3% decrease in the acquisition rate among 100 patients with CRGN bacilli during the ICU stay and with an 84% decrease in the risk of becoming a carrier independently of any other characteristic. Such a strategy has previously been reported to be of interest in numerous contexts [16, 17] and against various multidrug-resistant species [16, 18], although the benefit appears clearer for MRSA than for CRGN bacilli [16, 19]. In addition, numerous variations in the components and in the application of isolation precautions may also impact the efficiency of this strategy, leading to controversial results [20, 21]. For example, the efficacy of such a strategy has been questioned in the context of high compliance with hand hygiene, possibly associated with systematic body washing with chlorhexidine, or in the case of a low prevalence of MRSA [22, 23]. The fact that our intervention occurred during a high-prevalence period of microbial resistance may favor the good results observed in our study compared with those reported in other studies. Of interest, in our study, indicators of hand hygiene compliance support an increase in hand hygiene frequency during the intervention period. Whether this is directly linked to the intervention following reminders during the training sessions between the two periods or to the fact that patients were under contact precautions with signs on the room’s door is complex to disentangle. As previously shown, training and education may have increased awareness of antibiotic resistance [24], which was translated into practice, including hand hygiene frequency. The evaluation of the relative impact of a higher frequency of hand hygiene with respect to other interventions was impossible in our study. However, the paramount role of hand hygiene to prevent MDRO transmission has already been reported [25]. Mathematical models support the efficacy of each of the components of our program [26, 27].

We chose a before-after design for our study because it is pragmatic and easily implementable in our setting, despite its limitations. The “colonization pressure” by CRGN bacilli on admission was similar in both periods. The fact that the patients in the intervention period harbored more traditional risk factors for MDRO carriage and acquisition, such as invasive devices and higher exposure to antibiotics before and within the ICU, reinforces our findings. However, we found that having more days of exposure to antibiotics within the ICU was linked to a lower acquisition rate. This may be because total antibiotic exposure includes antibiotics potentially active against CRGN bacilli. We tested this hypothesis, but there was a lack of power due to the low number of patients treated with these compounds. Molecular biology of isolated CRGN bacilli was not performed to ascertain the transmission link between patients, and it is possible that a few of the acquired cases were in fact imported and not detected on admission owing to the suboptimal sensitivity of rectal swab screening. However, this under-ascertainment of carriers on admission is likely to be similar in both periods.

Finally, although our intervention proved to be possible and effective in the Vietnamese setting, the question of implementing this strategy in the long term or in other settings is not resolved. Indeed, there is currently no funding or national reimbursement for screening samples in Vietnam, and most middle- or low-income countries are struggling to prioritize other major health topics. Bringing more evidence of the efficacy of such interventions on antibiotic resistance may initiate changes in health policies. By decreasing the cost of screening, the use of locally developed screening plates may be an alternative to commercial plates [28] and may help convince stakeholders to initiate or pursue similar programs.

In conclusion, the prevalence of CRGN bacilli, including carbapenemase-positive Enterobacterales, is high in our referral center in Vietnam, as approximately one-third of patients are carriers on ICU admission. These findings confirm previous data indicating that antibiotic resistance is a major issue in Vietnam. In addition, the proportion of CRGN bacilli acquired within five distinct ICU was worrisome without intervention, despite emphasis on the role of hand hygiene in preventing hospital-acquired infections in the period following the COVID-19 pandemic. The implementation of a new search and isolation policy was efficient in decreasing the cross-transmission rates of these MDROs. The collateral increase in hand hygiene compliance likely played an important role in the positive results of the new policy. The long-term effect of the search and isolation strategy will be dependent on new funds for laboratory analyses.

## Supplementary Information

Supplementary Material 1.

## Data Availability

The datasets analyzed during the current study are available from the corresponding author upon reasonable request.

## Abbreviations

ABHR: Alcohol-based hand rub
AMR: Antimicrobial resistance
CPE: Carbapenemase-positive Enterobacterales
CPPA: Carbapenemase-positive Pseudomonas aeruginosa
CRAB: Carbapenem-resistant Acinetobacter baumannii
CRGN: Carbapenem-resistant gram negative
HH: Hand hygiene
HR: Hazard ratio
ICU: Intensive care unit
IQR: Interquartile range
KPC: Klebsiella pneumoniae carbapenemase
MCH-108: Military central hospital 108
MDRO: Multidrug-resistant organism
MRSA: Methicillin-resistant Staphylococcus aureus
NDM: New-Delhi metallo-beta-lactamase
OR: Odds ratio
OXA: Oxacillinase
SEA: Southeast Asia
95%CI: 95% Confidence interval
