# Supplementary material for: Evaluation of a screening and isolation strategy to curb carbapenem-resistant gram-negative bacteria in Hanoi, Vietnam: a pragmatic before-after study
Source: BMC Infect Dis. 2025 Oct 9;25:1264. doi: 10.1186/s12879-025-11736-2 (PMC12512652; doi:10.1186/s12879-025-11736-2)
Supplement: Supplementary file 1 — Supplementary Material 1. [file 12879_2025_11736_MOESM1_ESM.docx]

**Evaluation of a screening and isolation strategy to curb carbapenem-resistant gram-negative bacteria in Hanoi, Vietnam: a pragmatic before-after study**

By Toan Nguyen Quang, et al.

Table S1. Comparison of the characteristics of patients free of CRGN* bacilli on ICU admission in the reference and intervention periods

| **Characteristic** | **Reference** | **intervention** | ***P* value** |
| --- | --- | --- | --- |
| **Qualitative variable** | **310 (100)** | **302 (100)** |  |
| Gender (male) | 218 (70.3) | 199 (65.9) | 0.240 |
| Admission in ICU |  |  | 0.006 |
| - 1 | 70 (22.6) | 55 (18.2) |  |
| - 2 | 56 (18.1) | 75 (24.8) |  |
| - 3 | 79 (25.5) | 78 (25.8) |  |
| - 4 | 63 (20.3) | 36 (11.9) |  |
| - 5 | 42 (13.6) | 58 (19.2) |  |
| Patients’ origin before ICU |  |  | 0.001 |
| - Home | 38 (12.3) | 16 (5.3) |  |
| - Emergency room | 67 (21.6) | 73 (24.2) |  |
| - Other healthcare institution | 109 (35.2) | 140 (46.4) |  |
| - Other wards of MCH-108 | 96 (31.0) | 73 (24.2) |  |
| Prior hospitalization | 212 (68.4) | 221 (73.2) | 0.193 |
| Antibiotic before ICU | 97 (31.3) | 151 (50.0) | <0.001 |
| Invasive device in ICU-stay | 269 (86.8) | 289 (95.7) | <0.001 |
| - Mechanical ventilation | 248 (80.0) | 243 (80.5) | 0.886 |
| - Central intravenous catheter | 181 (58.4) | 200 (66.2) | 0.046 |
| - Urinary catheter | 267 (86.1) | 289 (95.7) | <0.001 |
| Hospital-acquired infection | 123 (39.7) | 62 (20.5) | < 0.001 |
| Antibiotic regimen in ICU | 273 (88.1) | 285 (94.4) | 0.006 |
| - penicillin | 29 (9.4) | 21 (6.9) | 0.441 |
| - beta-lactam + inhibitor | 32 (10.3) | 22 (7.3) | 0.185 |
| - extended-spectrum cephalosporin | 108 (34.8) | 79 (26.2) | 0.020 |
| - carbapenem | 161 (51.9) | 173 (57.3) | 0.184 |
| - newer antibiotics | 36 (11.6) | 71 (23.5) | <0.001 |
| - fluoroquinolones | 158 (51) | 117 (38.7) | 0.002 |
| - aminoglycosides | 88 (28.4) | 27 (8.9) | <0.001 |
| - colistin | 16 (5.2) | 7 (2.3) | 0.109 |
| CRGN-positive on ICU discharge | 196 (63.2) | 108 (35.8) | <0.001 |
| **Continuous variable** | **Median (25-75%)** | **Median (25-75%)** |  |
| Age (year) | 65 (51-76) | 65 (50-74) | 0.420 |
| Days in hospital before ICU | 1 (0-3) | 1 (0-3) | 0.851 |
| Total duration of antibiotic regimen (day) | 7 (4-9) | 9 (7-12) | <0.001 |
| Treatment duration with (days) |  |  |  |
| - penicillin | 0 (0-0) | 0 (0-0) | 0.472 |
| - beta-lactam + inhibitor | 0 (0-0) | 0 (0-0) | 0.359 |
| - extended-spectrum cephalosporin | 0 (0-3) | 0 (0-0) | 0.037 |
| - carbapenem | 1 (0-7) | 6 (0-10) | <0.001 |
| - newer antibiotics | 0 (0-0) | 0 (0-0) | <0.001 |
| - fluoroquinolones | 0 (0-6) | 0 (0-4) | 0.006 |
| - aminoglycosides | 0 (0-2) | 0 (0-0) | <0.001 |
| - colistin | 0 (0-0) | 0 (0-0) | 0.152 |
| * Carbapenem-resistant gram-negative bacilli | | | |

Table S2. Subgroup analyses of incidence density rates (per 100 patient-day) in the reference and intervention periods

| **Subgroup** | **Reference** | **Intervention** |
| --- | --- | --- |
| *Overall* | 196 / 2860 (6.85) | 108 / 3870 (2.79) |
| *Department* |  |  |
| 1 | 46 / 659 (6.98) | 17 / 683 (2.49) |
| 2 | 32 / 467 (6.85) | 23 / 930 (2.47) |
| 3 | 59 / 813 (7.26) | 40 / 1077 (3.71) |
| 4 | 31 / 531 (5.84) | 10 / 372 (2.69) |
| 5 | 28 / 390 (7.18) | 18 / 808 (2.23) |
| *Gender* |  |  |
| Male | 140 / 1935 (7.24) | 67 / 2490 (2.69) |
| Female | 56 / 925 (6.05) | 41 / 1380 (2.97) |
| *Age group* |  |  |
| ≤65 years | 103 / 1355 (7.60) | 48 / 1886 (2.55) |
| >65 years | 93 / 1505 (6.18) | 60 / 1984 (3.02) |
| *Origin before admission* |  |  |
| Home/community | 21 / 328 (6.40) | 4 / 186 (2.15) |
| Other hospitals | 71 / 942 (7.54) | 47 / 1735 (2.71) |
| Other wards of H108 | 62 / 1007 (6.16) | 25 / 937 (2.67) |
| Emergency room | 42 / 583 (7.20) | 32 / 1012 (3.16) |
| *Previous hospitalization* |  |  |
| Yes | 140 / 2020 (6.93) | 75 / 2788 (2.69) |
| No | 56 / 840 (6.67) | 33 / 1082 (3.05) |
